# Toward promoting performance nutrition behaviors among tactical athletes: a mixed methods study

**DOI:** 10.1080/15502783.2025.2492186

**Published:** 2025-05-02

**Authors:** Bradley Baker, Julia Carins, Rosa Peterson, Regina Belski, Matthew B. Cooke

**Affiliations:** a Food and Nutrition, Human Systems Performance, Defence Science and Technology Group, Department of Defence, Scottsdale, Australia; b School of Allied Health, Human Services and Sport, La Trobe University, Sport, Performance and Nutrition Research Group, Bundoora, Australia; c Swinburne University of Technology, School of Health Sciences, Hawthorn, Australia; d Griffith University, Social Marketing @ Griffith, Department of Marketing, Nathan, Australia

**Keywords:** Diet and nutrition, military, performance nutrition, behaviour change, relative energy deficiency in sport

## Abstract

**Background:**

Appropriate and adequate nutrition is essential for the health and performance of tactical athletes, including army recruits and trainees. However, previous research shows they commonly experience suboptimal intakes of energy and carbohydrate. To date, little research has been conducted to understand the causes of their poor dietary intakes. The objectives of this study were to (1) assess infantry trainees’ dietary intakes and (2) explore their beliefs, barriers, and self-efficacy toward performance nutrition using the Health Belief Model (HBM).

**Methods:**

Participants undergoing their 17-week Australian Army Infantry Initial Employment Training (Infantry IET) were recruited. During weeks 1 and 17, self-reported dietary intakes were assessed, and four focus groups were conducted.

**Results:**

Mean daily energy, carbohydrate, and protein intakes were 7864 ± 1135 kJ, 1.8 ± 0.6 g/kg, and 1.5 ± 0.3 g/kg, respectively, in week 1, and 9084 ± 2535 kJ/day, 2.2 ± 1.1 g/kg and 1.5 ± 0.2 g/kg, respectively, in week 17. Three themes and seven subthemes were developed from the focus group data, falling under the following HBM domains: Cues to Action, Perceived Barriers, and Self-Efficacy.

**Conclusions:**

Infantry trainees’ ability to eat well for their health and performance was hindered by their limited time during their intensive training schedule, their limited access to a sufficient variety of healthy foods, and their limited self-efficacy regarding performance nutrition. They require further performance nutrition knowledge, especially in regard to adopting practical fueling and refueling strategies surrounding physical training, as well as access to a greater quantity and variety of healthy foods outside of their main mealtimes.

## 1. Background

Australian Army recruits and trainees progressing toward a career as infantry soldiers undertake approximately 6 months of initial training to develop their skillset and physical and mental preparedness for front-line combat service. The initial training pathway commences with a 12-week Basic Training (BT) course, after which they graduate from recruit to trainee and complete a 17-week Infantry Initial Employment Training (Infantry IET) course. Infantry trainees can be considered as tactical athletes due to their high physical workloads, their need to maintain competitive performance on the battlefield, and the range of psychological and physiological stressors they experience [1,2]. Inadequate dietary intake has been identified as one of those key stressors in previous studies [1,2]. The Military Recommended Dietary Intakes (MRDIs) for energy are 11,500–13,000 for female trainees and 17,000–19,000 kJ/day for male trainees [3], and carbohydrate intakes of 6–8 g/kg/day are also required [4]. However, their energy and carbohydrate intakes have often been found to be well below these levels [5–7], contrary to guidelines published by the International Society of Sports Nutrition, which recommend they achieve adequate energy and macronutrient intakes to reduce injury risk and promote performance [8]. Further, among sporting athletes, low intakes of energy, or low energy availability (LEA), has the potential to impact adaptations to training and lead to Relative Energy Deficiency in Sport (REDs) syndrome and a wide range of adverse health and performance outcomes [9,10]. Due to their high energy demands and often inadequate dietary intakes, army recruits and trainees may be at similar risk of LEA and relative energy deficiency syndrome, termed Relative Energy Deficiency in the Military (RED-M) [11,12]. Similar to REDs, adverse outcomes of RED-M could include impairments in energy metabolism, musculoskeletal health, immunity, glycogen synthesis, and cardiovascular and hematological health [10,12]. For recruits and trainees, potential consequences include decreased fitness and performance, both in fitness assessments and in physically demanding aspects of their training and an increased risk of bone stress injury [13].

Military personnel and athletes alike often face a series of challenges in achieving performance nutrition guidelines, which may be a contributing factor to LEA [7,14]. Among infantry trainees, there is a paucity of studies that have explored these challenges, with some suggesting it to be multifactorial in nature and be linked to a combination of increased physical demands and extremely busy training

schedules [15]. Thus, it is important to understand the perceptions of Australian infantry trainees, as well as other potential drivers of their eating behaviors, such as their self-efficacy and motivations to eat well for their health and performance.

The Health Belief Model (HBM) is a model of behavior comprised of six domains, with these being 1) perceived susceptibility to the problem, 2) perceived severity of the problem, 3) perceived benefits, 4) perceived barriers and enablers, 5) self-efficacy, and 6) cues to action. It has previously been used to underpin focus group research to systematically explain why groups may or may not engage in particular behaviors [16–19] and can be used to gather evidence to inform behavior change interventions aimed at improving dietary intakes and performance [14]. To our knowledge, no studies to date have systematically investigated the perceptions, barriers, enablers, self-efficacy, and cues to action toward eating well for health and performance among Australian infantry trainees. By concurrently assessing trainees’ dietary intakes and highlighting areas where their motivators, barriers, beliefs, and self-efficacy may be contributing to their poor dietary intakes, this study will identify HBM domains under which behavior change strategies can be formulated. Thus, the objectives of this study are to (1) assess the dietary intakes of infantry trainees undertaking Infantry IET and compare these to their requirements (2) explore the HBM domains that may explain infantry trainees’ ability to eat well for health and performance.

## 2. Methods

### 2.1. Participants

Voluntary participation was sought from Australian Army infantry trainees (*n* = 48) during Week 1 of Infantry IET at Lone Pine Barracks, Singleton, New South Wales, Australia, in February 2022. This involved the delivery of a verbal brief about the study, followed by the distribution of a Participant Information Sheet and a Consent Form. Informed consent was sought by asking those wishing to volunteer to read the information and complete the Consent Form along with a Health Questionnaire, all in the absence of any more senior Australian Army members with whom potential participants would be in an unequal relationship with. Volunteers (*n* = 17) were screened and considered eligible if they did not report a history of a diagnosed eating disorder in the Health Questionnaire. All 17 volunteers were recruited to the study, participated in the four focus groups across weeks 1 and 17 of Infantry IET, and were included in the thematic analysis. Ten of the 17 participants completed their 17-week Infantry IET within the study period and completed all six 24-h diet recalls across weeks 1 and 17, and these 10 participants were included in the statistical analyses. Each week of Infantry IET is characterized by high physical demands and daily physical training (PT) with a Physical Training Instructor (PTI), including weeks 1 and 17. Ethics approval to conduct the study was granted both by the Defence Science and Technology Group Low Risk Ethics Panel (protocol LD 03–20) and the Swinburne University Human Research Ethics Committee (protocol 20,202,880–3968).

### 2.2. Self-reported dietary intakes

On three consecutive training days (Tuesday, Wednesday, and Thursday) during Infantry IET weeks 1 and 17, participants completed the online Automated Self-Administered 24-Hour Australia (ASA24-Australia 2016) Dietary Assessment Tool [20]. The ASA24-Australia asked respondents to enter all of the meals, snacks, and drinks they had consumed on the previous day (from 12:00 am to 11:59 pm). Participants’ daily energy intakes were compared to their MRDI of 17,000 kJ/day, which is based on an energy expenditure study using the doubly labeled water method among Australian Infantry trainees [3]. Participants’ carbohydrate and protein requirements were compared to the lower end of their MRDIs of 6 g/kg/day and 1.5 g/kg/day, respectively [3,4].

### 2.3. Statistical analyses

Hypotheses were specified, and statistical analyses were planned, prior to the commencement of participant recruitment and data collection. All statistical analyses were performed using the Statistical Package for the Social Sciences (SPSS) version 25 [21]. No outliers were identified in participants’ dietary intakes of energy and macronutrients, as assessed by inspection of boxplots. The distribution of differences in dietary intakes of energy and each macronutrient between weeks 1 and 17 was determined to be normal using the Shapiro–Wilk test using a *p* value of <0.05. A series of paired t-tests were conducted to determine any significant differences between dietary intakes of energy and each macronutrient at weeks 1 and 17. The level of statistical significance was set at *p* < 0.05. All demographic and dietary intake data are presented as mean ± standard deviation.

### 2.4. Focus groups

Four focus groups, lasting up to 45 min, were conducted following the practical guide by Greenbaum [22], with two in each of weeks 1 and 17 of Infantry IET. These were moderated by one author, an Accredited Sports Dietitian with Sports Dietitians Australia (B.B.; Bradley Baker), in a private room with only the participants and researchers present. At the beginning of each focus group, participants were introduced to the moderator and note taker and briefed about what the session would involve. This included the purpose of the focus groups, the anonymity of all responses, the use of audio recording and the topics that would be discussed.

Focus groups were moderated using a semi-structured discussion guide, beginning with a warm-up question and following with questions underpinned by each domain of the HBM. The discussion guide (shown in Supplementary Table S1) included potential prompts and shows how the HBM was used to explore factors related to eating well for health and performance during Infantry IET [16].

### 2.5. Thematic analysis

As preplanned, the abridged transcripts of focus groups were thematically analyzed based on the 6-phase approach described by Braun and Clarke [23]. This approach has been described as a theoretically flexible research method that is able to be used in either an inductive or deductive manner [23]. This study blended both inductive and deductive approaches – inductively generating themes to capture the ideas of the participants and deductively exploring the data within the framework of the HBM. The foundations of our approach are our relativist ontology and constructivist epistemology. As a group of sports dietitians, nutritionists, and nutrition researchers, we have a background in helping individuals and populations overcome barriers to eating well, and in applying evidence-based performance nutrition guidelines in research and practice. One author (B.B.) was involved in phases 1–4 of thematic analysis, which were conducted manually in Microsoft Word. In phase 1, the audio recordings were carefully listened to and transcribed into an anonymous abridged transcript, and ideas related to the study aims were written down. In phase 2 (generating initial codes), short and succinct codes were assigned to all data extracts. The generation of initial themes (phase 3) involved clustering connected codes into candidate themes. In phase 4 (developing and reviewing themes), candidate themes were assessed against both coded data extracts and the full dataset for their appropriateness in conveying the pertinent common themes and adjustments were made accordingly. Phase 5 (refining, defining, and naming themes) and phase 6 (writing up) involved all authors reviewing, refining, and writing up the themes in the manuscript [23].

## 3. Results

### 3.1. Demographics

All volunteers were male infantry trainees, with an average age: 22.6 ± 2.7 years, height: 182 ± 5.6 cm, and weight: 86.5 ± 10.0 kg.

### 3.2. Self-reported dietary intakes

Participants’ mean self-reported dietary intakes of energy and each macronutrient are shown in Table 1. Participants’ mean energy intakes met 46% and 53% of their estimated energy requirement of 17,000 kJ/day during weeks 1 and 17 of Infantry IET, respectively. Relative to their body weight, participants’ carbohydrate intakes (mean ± SD) were 1.8 ± 0.6 g/kg/day and 2.2 ± 1.1 g/kg/day during weeks 1 and 17, respectively. All participants reported carbohydrate intakes well below the lower end of their requirement of 6 g/kg/day. Their protein intakes (mean ± SD) were 1.5 ± 0.3 g/kg/day and 1.5 ± 0.2 g/kg/day during weeks 1 and 17, respectively. During weeks 1 and 17, 40% and 30% of participants reported protein intakes <1.5 g/kg/day, respectively.

**Table 1.** Dietary intakes of energy and macronutrients at weeks 1 and 17 of infantry initial employment training.

Dietary intake	Week 1 (Mean ± SD^a^)	Week 17 (Mean ± SD^a^)	*p* Value^b,c^
Energy (kJ)	7864 ± 1135	9084 ± 2535	.15
Carbohydrate (g)	156 ± 48	175 ± 75	.44
Protein (g)	135 ± 25	128 ± 36	.59
Fat (g)	76 ± 15	102 ± 33	.01^3^

^a^SD = Standard deviation.

^b^*p* values were calculated by paired *t*-tests.

^c^*p* values < 0.05 were considered significant.

^d^Intakes as a percentage of the lower end of the MRDIs for energy, carbohydrate and protein.

As shown in Table 1, participants’ total dietary intakes of energy, carbohydrate, and protein were not significantly different between weeks 1 and 17. Their total fat intakes were significantly higher at week 17 compared to week 1 (∆ 26 ± 27 g/day; 95% CI, 7 to 46).

### 3.3. Focus groups

All domains of the HBM were visible in the data, with agreement among many participants that they were susceptible to the problem, expressing sentiments that indicated they were unable eat well enough to optimize their performance and mitigate their risk of suffering from adverse health outcomes throughout their Infantry IET. Also, many participants conveyed that the problem was severe, being a frequent occurrence that they were unable to either eat enough or eat enough of the right foods during their Infantry IET. Moreover, many participants described several potentially severe consequences of not eating well enough during their Infantry IET, including early fatigue, muscle atrophy, and injury. Below, three key themes and six subthemes were constructed from the data, with these falling under three HBM domains, as shown in Table 2. Each theme is summarized with supporting quotes in-text below.

**Table 2.** Themes and subthemes under health belief model domains.

HBM Domain^a^	Theme	Sub theme
Cues to Action	Motivators: Performance, Fuel Needs and Preventing Fatigue	Performance and Fitness Goals Fuelling Performance and Preventing Fatigue, Muscle Atrophy and Injury
Perceived Barriers	Limited Time and Access	Limited Time to Eat Enough Time-Limited Mealtimes within Intensive Training Schedules Limited Access to a Sufficient Variety of Foods Outside of Main Mealtimes
Self-Efficacy	Limited Performance Nutrition Knowledge	Limited Procedural Knowledge on Fueling and Refueling Strategies Limited Education on Performance Nutrition

^a^HBM = Health Belief Model.

#### 3.3.1. Domain:cues to action

Motivators: performance, fuel needs, and preventing fatigue

##### 3.3.1.1. Performance and fitness goals

Across all focus groups, participants described the importance of eating well to perform well in both their role as infantry soldiers-in-training and in their physical training (PT) sessions. Participants expressed that the large amount of physically demanding PT they undertake (approximately 2 h each weekday) and the additional physical demands placed on them in their job were their key motivators to eat well. Participant 12 described this in the extract below:

Moderator: “What’s the main reason you want to eat better? What motivates you to eat better?”

Performance, you want to perform well. Perform to your best, so eat well. … It’s probably one of the most important things at the school, is to eat. Especially with the amount of PT and loading and everything we do. You’ve got to eat even better, you know.

In another focus group, participant 14 described a similar perspective, adding that their job requires them to be on their feet all day, with other participants agreeing with him:

Just the amount of PT we do, I guess. You just know that we’re always on our feet … the PT sessions are not easy obviously, and then we’re always on our feet all day, so you just know: I’ve just gotta eat as much as I can.

Across the focus groups, participants’ primary fitness goals and their associated motivators to eat well differed. For example, participant 8 reported his main motivation for eating well was “just to get bigger. Simple as that,” whereas other participants reported that improving their physical fitness more holistically was their main motivator. For example, participant 13 stated:

You know, we do lots of physical activity, [so] you want to sort of be, not really bulking, but like improving your fitness as well.

##### 3.3.1.2. Fuelling performance and preventing fatigue, muscle atrophy, and injury

Participants described the fuel needs that are associated with their high physical demands as their main motivators. Participant 6 described this:

My motivation … is pretty much like: the more you put in, the more you put out, and just sending it every day at PT, and needing that fuel to actually be able to send it. The more you put in, the more you put out.

Similarly, in the extract below, participant 4 described his experience of feeling fatigue kick in earlier during PT sessions when he had not eaten well beforehand:

Moderator: “In terms of the amount of physical activity you do, your training sessions, your training adaptations … what are some of the consequences that could occur if you don’t eat well?”

I mean you can feel the fatigue kick in, or like that little voice in your head a lot earlier that’s telling you to stop when you haven’t eaten well, or haven’t prepared well for that [training] session.

There was broad agreement among participants about the risk of becoming tired, fatigued and not being able to keep up with fellow trainees, particularly following PT sessions, if they hadn’t eaten well enough to adequately fuel and refuel. In the extract below, participant 13 described this, with several fellow participants voicing their agreement:

Moderator: Suppose you don’t eat well, you eat badly, you’re not eating optimally at all – what are some of the consequences that you think can happen?

You can’t perform as well … just not being able to perform as well as you’d like to. Like if you’re super tired [and] knackered after a PT session, then you won’t be able to keep up with the rest of the crew … you’ve just gotta try and like, make sure you get enough energy.

Continuing on this topic, participant 13 described his tendency to start to feel fatigued and fall asleep when he had not been able to eat enough at each main meal throughout the day:

Ohh, especially in lessons, you fall asleep … if we’re sitting in class in the afternoon … you just feel yourself dozing, because you … tried to eat a big lunch and couldn’t fit it all.

Also, on this topic, participants described several potential and severe consequences of not eating well enough during their Infantry IET. Participant 16 described injury as a potential consequence, with other participants agreeing with him. Following on from participant 16, participant 6 and described muscle atrophy as a severe consequence.

### 3.4. Domain: perceived barriers

#### 3.4.1. Limited time and access

##### 3.4.1.1. Limited time to eat enough

Across all focus groups, participants discussed having limited time to eat enough food during their busy training schedule. In the extract below, participant 4 shared his perspective on what the main barriers to good nutrition are during Infantry IET, with limited time to eat enough food being the biggest limitation:

Time is a big one … That’s probably the biggest limitation in my eyes on like adequately eating enough food.

Participants described having limited time to eat breakfast in the morning, both before commencing their morning PT session as well as after completing it. Further, participants reported feeling unsatisfied with the amount they can eat at breakfast (due to having limited time amongst the demands of their training program, such as attending lessons and PT sessions). For example, participant 14 stated:

Mainly [having too little time for] breakfast is just the worst one [out of breakfast, lunch and dinner]. Like, even yesterday, we had like, what was it, like 5 minutes [after PT] to get dressed, smash down [an egg and bacon roll] and then we were back at lessons like straight away.

In congruence with this, participant 4 perceived that there was “no consistency” in his time and opportunity to eat a substantial breakfast between waking up, attending his PT session, and then undertaking subsequent training.

##### 3.4.1.2. Time-limited mealtimes within intensive training schedules

Throughout the discussions, many participants shared the perspective that the mess/dining hall was only open for a limited time and only over the three main mealtimes. A number of participants expressed the challenge they face with trying to eat particularly large meals in order to meet their needs within their given meal breaks, which must fit within their intensive training schedule. Participant 16 expressed this in the extract below.

I typically would prefer to have, you know, small meals throughout the day—obviously it’s not appropriate in this situation. So, I find it hard to have three huge meals.

Several other participants also described the challenge of trying to eat very large meals when in the mess/dining hall. For example, participant 13 reported often being unable to “fit” in all of his lunch, but then suffering the consequence of “dozing” off in the afternoon during lessons if he had not been able to eat enough at lunch to keep himself feeling sustained.

In regard to dinner, participants described their discontent that the mess/dining hall opens relatively early and closes shortly thereafter and that they are extremely hungry again 1–2 h after eating dinner. Participant 12 stated:

Our timing for dinner is 4:45 … and then [at] 6:15 they close … and then we don’t go to bed until 10 o’clock … by the time you go to bed you’re starving again. [Fellow trainee] and I are definitely hungry again an hour, two hours later [after eating dinner], and you’re just sitting in bed [with a stomach] growling and you feel sick when you get up in the morning.

Participants shared their perspective that although they are provided with snack foods that are accessible between scheduled mealtimes in the mess/dining hall and throughout the evening, they sometimes lack the time or ability to access them (e.g. during field training). For example, participant 13 stated:

There’s plenty of [snack food] options there but it’s just like whether or not we get the time of day to go back [to our accommodation building] and grab them. Unless we’re … if we’re out on the range obviously we don’t have access to that or anything like that.

##### 3.4.1.3. Limited access to a sufficient variety of foods outside of main mealtimes

Participants considered many of the snack foods they are provided to be high in sugar. Furthermore, they stated that choices of healthy and substantial snacks and light meals were limited outside of the three main mealtimes in the mess/dining hall. Participant 12 stated, in regard to these foods, that they are “nothing they class as healthy options.” Additionally, participant 16 stated:

There’s a lot of sugar in the snacks that we get, that’s my only thing. It’s like, should I be eating this much sugar? I mean, I can get away with it, because I’m burning so many calories, but …

Participant 1 described his perception of a limited variety of healthy, non-processed choices for more substantial foods, with a limited range of healthy and filling foods that can be eaten as either a light meal or as a meal in situations when their training schedule had imposed on their mealtime:

Say if we were hungry, probably the go-to would be the scroll, [in the common room of our accommodation building] we [were provided with] cheesy-mite, vegemite scrolls—we put them in the microwave. That was the only real filling food we have. So, if you couldn’t go to the mess at night or whatever happened, you’d go and have a couple of scrolls, but it’s not a very healthy [snack] … to have all the time, I would say, is not the best situation. Ideally, there could be some better, more filling foods available.

During focus group discussions, many participants described their frustration at being unable to eat well due to having limited access to a variety of healthy and substantial foods outside of main mealtimes in the mess/dining hall. They described their need to regularly buy foods from either the on-base takeaway shop or to order pizza from the nearby town to satisfy their hunger and feel sustained (as these are the only accessible and affordable options available to them), while, at the same time, actively trying to avoid these foods because they are unhealthy. In the extract below, participant 12 described this situation.

Moderator: “And so, throughout the evening, there’s not enough good, healthy foods available, that’ll keep you feeling full and sustained?”

Yeah, you can go and get a burger and chips [from the on-base takeaway shop] but you don’t wanna do that, you know? Or you do, but you don’t. … [Burgers are] good every now and then, but you don’t want a burger every day.

In the extract below, participant 3 also described the limited healthy choices he and his fellow trainees have for more substantial foods outside of main mealtimes in the mess/dining hall, particularly in the evening:

There’s not really much good food you can have from [on-base takeaway shop] as a second option. Like, you might have a few sandwiches that are reasonably healthy throughout the day, but everything else is fast fatty food, and yeah it’s just standard—you don’t really feel good after eating it. … [Ordering] pizza [from the nearby town and getting it delivered] was a massive thing that we had to do when we didn’t have time for dinner.

Many participants shared a similar perspective that they are sometimes unable to attend the mess/dining hall to eat meals due to their training commitments, or that sometimes their time and/or opportunity to eat main meals can be limited due to their training schedule. As a result, they expressed that they rely on fast food that is often fatty and unhealthy such as pizza (as expressed by participant 3 above).

Participants also described having a reliance on vending machines for snacks to satisfy their needs and keep themselves feeling awake, particularly following main mealtimes in the mess/dining hall in the mid-morning and mid-afternoon during lessons. However, they described that the food available in vending machines is junk food and suboptimal for their needs. For example, participant 9 stated that in order to eat enough food, he and his fellow trainees:

… have to stack up with like, vending machines type of [junk food], [the on-base takeaway shop] a lot.

Participant 13 also described a reliance on vending machines: “you go [to the vending machine], you can get like an energy drink and you’ll stay awake for hours … which is what you need sometimes.”

### 3.5. Domain:self-efficacy

#### 3.5.1. Limited performance nutrition knowledge

##### 3.5.1.1. Limited procedural knowledge on fuelling and refuelling strategies

Focus group discussions revealed varied levels of knowledge and understanding of the role of carbohydrate intakes in fueling endurance and performance during moderate-to-high intensity training. In the below extract, participant 12 described the importance of increasing his carbohydrate intake before a Basic Fitness Assessment (BFA) to ensure he has sufficient energy stores, given that sufficient energy (glycogen) stores equate to prolonged physical endurance:

Moderator: “Okay so changing topics a little bit, what about carbohydrate? What do you guys know about carbohydrate?”

Yeah, so, you know, like before a BFA or whatever, we get told, you know, carb load, give yourself HEAPS of energy stored up, so when you hit that like … that threshold, you can still have backup, like boost, just to go.

However, despite some participants having declarative knowledge of the importance of carbohydrate intake in enhancing performance and prolonging endurance, many participants displayed limited procedural knowledge in the application, especially regarding eating strategies that can be utilized immediately before, during and after PT sessions to promote adequate fueling and refueling. Moreover, across all focus groups, participants displayed an apparent lack of knowledge of which foods and drinks provide rapidly digestible carbohydrate and thus reduce the risk of suffering from gastrointestinal issues (e.g. vomiting) when eaten immediately before and during moderate-to-high intensity physical activity. This was highlighted in the following discussion extract involving participants 13 and 4:

Participant 13: “In the mornings, you know, if we go to the mess or whatever and we have a big hearty brekky [and] make sure we’re, you know, fueled for the day, we’ll go to a PT session with [a Physical Training Instructor] and we’ll just vomit it all up, so it doesn’t end up staying down for very long.”

Participant 4 then stated: “It’s like a trade off on those mornings, you’ve really got to be like: What will I eat today? What is going to stay inside me?”

The moderator prompted these participants to share any knowledge they had about what foods and drinks are suitable immediately before intense physical activity to reduce the risk of suffering from gastrointestinal issues. In the extract below, their procedural knowledge gaps on this topic were reinforced:

Participant 4: “It’s more like us talking among each other and being like ohh yeah, not … We don’t have like a direct nutritionist that comes and is like ‘if you’ve got this lined up … ’ it’s more just word of mouth.”

Further, participant 5 described being in the habit of not eating breakfast to reduce his risk of vomiting during his subsequent PT session, and described his observation that his fellow trainees are in the same habit:

I’ve noticed at breakfast, people get into a habit, myself included, to not eat before, or not eat breakfast at all. Because we might have a PT session straight after, and a lot of people will just puke up their guts … and you know, you don’t want to puke up your guts, so people will just take the option of not eating breakfast, and doing the PT, and then buying something from [the on base takeaway shop] later on or something like that, because you don’t want to taste your breakfast twice.

Participants shared having received a nutrition brief from their PT instructors at the start of their IET, with many acknowledging that this had played a pivotal role in their understanding of the importance of good nutrition throughout their IET. For example, participant 4 stated: “At the start of our PT program … We’re told that nutrition is important.” However, participant 4 perceived that gaps remained in his procedural understanding of how to achieve good nutrition. For example, he explained that, although he’d been told that nutrition is important, he didn’t know “specifically what aspects of nutrition are important.” When prompted again about what foods or drinks participants knew of that they could eat immediately before vigorous activity to reduce their risk of suffering from gastrointestinal issues, participant 4 explained that he didn’t know what foods to avoid, with participant 13 explaining that is understanding was limited to: “ … don’t eat too much [beforehand] because it’ll come up.”

##### 3.5.1.2. Limited education on performance nutrition

Participants were asked whether they would benefit from being provided with greater education on ways to eat before and after their training that can promote performance and recovery, with favorable responses across all focus groups. In the extract below, participants 13 and 14 discussed some of the reasons they thought they would benefit from this.

Moderator: And so, more specific tips into what foods, when, how … would those sort of things be helpful?

Participant 13:“Yeah. ‘cause … the (staff who instruct us) told us that as (staff who instruct us) they’re actually not allowed to recommend [Participant 4: Foods], like, they’re not qualified nutritionists, so they can’t tell us what to eat, what not to eat. But they can say, you know, make sure you eat plenty of good food, drink plenty of water … .”

Participant 14: “Yeah. They can’t tell us like “don’t eat this at the mess, or “don’t eat that at the mess, eat this!” … But like, they could try and give us a bit of insight, they expect us to like obviously get the knowledge ourselves and reach out and like get an understanding about it. … So, that (greater performance nutrition education) would help a lot.

Participants 13 and 14 also shared the perspective that they and their fellow trainees would benefit from having access to someone who is qualified in providing performance nutrition guidance:

Moderator: … and so do you think there would be any benefit in having someone, who is qualified, come in and provide that knowledge – do you think that’s something that would be worthwhile?

Participant 13: Like, a lot of people would benefit from it [Participant 14: Yeah!] … I think having someone there who can you know, show them like what to eat after doing certain things would really help, in terms of like staying fit, or you know, like gaining weight if they wanna gain weight [Other participant 14: Yeah] or lose weight, that sort of thing. Makes it much easier to have someone there to coach you through it.

## 4. Discussion

The current mixed-methods study found that trainees were motivated to eat well to help achieve their fitness goals and body weight goals, to improve their performance, and to prevent fatigue. Moreover, good nutrition was recognized as an important factor in supporting training adaptations and preventing musculoskeletal injury during Infantry IET. Despite this, a number of key barriers were identified that may explain their suboptimal dietary intakes during their highly demanding military training course. These included perceived lack of time and limited access to a sufficient variety of foods outside of main mealtimes, as well as lack of self-efficacy and procedural knowledge, particularly on eating strategies in the lead up to demanding PT. The self-reported dietary intake results are likely to represent an underestimation of participants’ actual energy and macronutrient intakes during training and thus, should be interpreted with caution. Notwithstanding, the perceived barriers, motivators, self-efficacy, and beliefs of trainees identified in this study should be considered when designing future strategies to improve dietary intakes and reduce the risk of REDs/RED-M and adverse health and performance outcomes.

Self-efficacy refers to one’s confidence in their ability to execute certain behaviors in order to achieve an outcome [24]. In the context of performance nutrition for infantry soldiers, it refers to their confidence in their ability to make the best food choices in order to achieve their best performance, both physically and cognitively, even when faced with potential barriers. In the current study, trainees’ self-efficacy appeared to be impeded by limited performance nutrition knowledge, especially in regard to foods and drinks that are suitable to be eaten prior to moderate and/or high intensity aerobic activity. Extensive research in athletic populations has led to the recommendation that foods high in carbohydrate should be eaten in the 3–4 h before moderate and/or high intensity aerobic activity to provide sufficient fuel [4,25–28]. Immediately before (<1 h) and during prolonged moderate-to-high intensity aerobic activity, foods that are high in carbohydrate and are rapidly digestible should be eaten to top up muscle fuel stores and also alleviate the risk of suffering gastrointestinal issues during training [4,27]. The current findings are similar to those of a recent study conducted among British Army recruits, which found participants had limited knowledge on eating carbohydrates for performance and recovery through administering the Nutrition Knowledge Questionnaire for Athletes [29]. In the current study, trainees were young male adults and reported having obtained much of their nutrition knowledge from their high school education. With no further nutrition education during BT or Infantry IET, especially in relation to supporting health and performance during military training, it is understandable that their nutrition knowledge is underdeveloped and lacking to meet their needs.

Skipping breakfast entirely, or consuming a very small amount of food, before morning PT sessions was a commonly reported habit among trainees in the current study. Their main reason for this was to avoid potential gastrointestinal (GI) issues during any moderate- or high-intensity aerobic activity. It is likely that trainees’ limited knowledge of suitable high-carbohydrate food and drink options, those which carry a low risk of GI upset and thus are appropriate for consumption before exercise, are contributing factors to this habit. A recent survey conducted among Australian Army officer cadets also found skipping breakfast was common, with 65% reporting that they never eat breakfast [30]. In an army initial training environment, routinely skipping breakfast before PT, coupled with having few and time-limited opportunities to eat over the remainder of the day, could place trainees at risk of LEA. As previously discussed, if prolonged over time, LEA may lead to REDs/RED-M [10,12], and associated decrements in health and performance, as demonstrated in studies conducted among sporting athletes [6,9,10,31,32]. Athletic and military guidelines recommend carbohydrate fueling in the lead up to all, or the vast majority, of training sessions to maximize training quality and performance [3,4,33].

A lack of access to a sufficient variety of foods outside of main mealtimes in the mess/dining hall was also perceived to be a barrier. In particular, a lack of choice for substantial options that are affordable, healthy, fresh, minimally processed, and low in added sugar and fat. Trainees shared the perspective that they often rely on high-fat fast foods outside of main mealtimes in the mess/dining hall, such as pizzas and burgers, which they can either order to be delivered to the base, or can purchase from the on-base canteen. In alignment with these qualitative findings, there was a significant increase in trainees’ fat intake at the end of their Infantry IET. Throughout Infantry IET, foods are typically provided to trainees across three main meals in the mess/dining hall (making ~17,000 kJ available per person per day), and in addition, snack foods (mostly shelf-stable; stored in the common room of trainees’ accommodation building) provide the equivalent of ~ 4,000 kJ per person per day. While a variety of healthy options are available during main mealtimes, trainees reported eating until they were completely full during their designated mealtimes at lunch and dinner, but then feeling extremely hungry again within 1–2 h, and consequently not feeling sustained until their next mealtime.

Further, trainees perceived that many of snack foods provided to them are high in sugar or aren’t healthy or substantial, and thus would often avoid them. These findings suggest that further designated snack and/or light meal breaks in the mess/dining hall, such as morning tea and/or supper, should be implemented whenever practicable throughout the intensive IET schedule, and changes to designated mess/dining hall opening times may alleviate trainees’ poor dietary intakes. This recommendation is supported by trainees’ perspective that the total quantity of foods provided during existing designated main mealtimes did not limit their ability to eat well for health and performance. Rather, they perceived a lack of sufficient time to eat enough healthy and substantial foods during existing main mealtimes in the mess/dining hall, and a lack of sufficient access to, and opportunity to eat, enough of the right foods outside of main mealtimes in the mess/dining hall. Their perceptions align with the on-base Infantry IET food environment, where trainees don’t have the freedom to purchase or store a large variety of healthy foods. Trainees reported feeling tired, unfocused and lethargic due to high hunger levels between meals, particularly in the mid-afternoon and mid-evening, and therefore these times would be ideal opportunities for providing trainees with additional access to a variety of fresh foods by offering additional mess/dining hall opening times. This may reduce their reported reliance on fast foods and ultra-processed foods and drinks, such as those from takeaway shops and vending machines, while improving the nutritional quality of their diet, better enabling them to meet their dietary preferences and requirements. Indeed, recent studies have demonstrated improved training adaptations and reduced injury rates as a result of better enabling army recruits and trainees to meet their nutritional requirements [5,34]. Thus, optimized nutrition-related policies and practices may be a cost-effective means of reducing the incidence of REDs/RED-M and improving trainees’ health, performance and training outcomes.

It was evident that the self-reported dietary intake data in the current study was far below participants’ estimated requirements and thus, is likely to represent an underestimation of their actual energy and macronutrient intakes. The ASA-24 has been shown to estimate intakes of energy and macronutrients with accuracy in a civilian population [35]. However, the current study was conducted during a demanding and time-intensive military training course, which may have led to the underestimation of participants’ energy and macronutrient intakes. In a laboratory setting, the completion of a 24-hour diet recall was shown to result in an underestimation of actual energy intake by up to 21% [36], and with the added stressors of the military training course, the underestimation of energy intake in the current study may have been even greater. Further, the method of weighing and recording all food selections and discards is considered as the gold standard in military settings and should be employed in place of diet recalls where feasible [37]. Together with more rigorously assessing dietary intake, consideration should also be given to including measures of dietary variety and of the distribution of energy and macronutrient nutrient intake across each day. The inclusion of these measures would provide a more comprehensive understanding of how improvements may be made. Notwithstanding these limitations and the inherent limitations of diet recall data in military settings, the findings of the current study do align with previous research indicating inadequate energy and carbohydrate intakes among army recruits and trainees [7]. In addition, the perspectives shared by participants in the current study indicated that they frequently felt unable to eat well enough to meet all of their nutritional needs.

The sample size sought in the current study was one that provided information-rich cases on beliefs, barriers and self-efficacy toward eating well, and guidelines for qualitative research indicate that 12 participants can be sufficient to reach data saturation [38]. Thus, the sample was suited to pursuing the research aims of this study, however we acknowledge that the sample size and composition of the current study does not support generalization to a broader population. Further, we acknowledge that selection bias may have influenced the findings, for example, infantry trainees who are more motivated and knowledgeable about eating well for their health and performance may have opted to participate. Lastly, nutrition knowledge for sport and exercise was not quantitatively captured, and therefore we are unable to more comprehensively characterize participants’ nutrition knowledge, and thus this is another limitation of the study.

## 5. Conclusions

In conclusion, infantry trainees’ self-reported intakes of energy and carbohydrate were well below the recommended levels, and thus, may place them at higher risk of the adverse health and performance outcomes associated REDs/RED-M. Trainees’ ability to eat well in accordance with performance nutrition recommendations is hindered by their intensive training schedules, their limited opportunities for mealtimes in the mess, as well as their limited access to a wide variety of foods, especially healthy options outside of main mealtimes. In addition, their lack of performance nutrition knowledge may further compound their perceived barriers to good nutrition. Although there can be limited flexibility in meal times due to the demanding and time-intensive nature of Infantry IET, improved performance nutrition knowledge among trainees, combined with greater access to fueling foods could improve their energy and carbohydrate intake, especially surrounding arduous physical activity. It is recommended that future behavior change strategies within army recruits and trainees should consider i) nutrition education focussing on performance nutrition (declarative and procedural), including practical fueling and refueling strategies before, during and after physically demanding training, ii) additional access to the mess/dining hall, such as in the mid-morning and/or evening, and iii) increased access to suitable foods and drinks prior to and during morning PT sessions to enable high carbohydrate intake and rapid absorption of carbohydrate to facilitate proper fueling. These strategies should be investigated for their efficacy in improving health and performance outcomes and preventing the incidence of problematic LEA.

## Supplementary Material

Supplemental Material
