# Supplementary material for: Toward promoting performance nutrition behaviors among tactical athletes: a mixed methods study
Source: J Int Soc Sports Nutr. 2025 May 2;22(1):2492186. doi: 10.1080/15502783.2025.2492186 (PMC12051564; doi:10.1080/15502783.2025.2492186)
Supplement: Supplemental Material [file RSSN_A_2492186_SM2427.docx]

## Supplementary online material

Supplementary Table. Focus Group Discussion Guide following each Domain of the Health Belief Model

| **Warm-up** |
| --- |
| 1. What are your thoughts on the food provided during your training? |
| **Perceived susceptibility to the problem** |
| 1. How confident are you that you can eat in the right way to meet your needs and perform at your best?   Potential prompts:   - What makes you this confident? - Why not more confident? - What reasons or factors make you more likely to have a suboptimal diet for physical during your training? - What foods are you lacking in to better support your physical abilities and health?  1. Considering the amount of physical training you do and your nutrition needs, how likely is it that you won’t eat optimally during your training?   Potential prompts:   - Why this likely?  1. How likely is that your diet isn’t optimal for your health or the development of your fitness or and strength?   Potential prompts:   - Why this likely?  1. How confident are you that you can eat in the right way to meet your needs and perform at your best? |
| **Perceived severity of the problem** |
| 1. Suppose you have a suboptimal diet and don’t meet your needs during your training program. How serious do you think the impact of this would be?   Potential prompts:   - What impacts would occur? - Giving an example of an impact to the group: how serious would the impact of this be?  1. Again supposing you have a suboptimal diet and don’t meet your nutritional needs during your training program. How serious would the impacts be on your physical abilities?   Potential prompts:   - What would be the impact on your fitness and strength gains if you don’t have an adequate diet? - What would be the impact on your strength gains if you don’t have an adequate diet? - What about the impacts of a suboptimal diet on your recovery from physical training. What impacts could occur? - What about the impacts of a suboptimal diet on your gains in muscle and strength. What impacts would occur, and how serious would these be? - What about the impacts of a suboptimal diet on your health – such as bone health and your risk of injury? In your opinion, how serious would the impacts of a suboptimal diet be on these? |
| **Perceived benefits** |
| 1. To what extent does your diet play a role in your ability to perform at your best during physical activity?   Potential prompts:   - According to you, are there ways in which you can eat to give you energy and improve your ability to perform at your best during exercise? Are any foods useful for exercise/physical performance? - To what extent does nutrition play a role in performance during a physical training program?  1. To what extent does your diet play a role in your health and recovery when you regularly exercise?   Potential prompts:   - What are some of the benefits of an optimal diet on your health and recovery when you regularly exercise? - To what extent does nutrition play a role in recovery during a physical training program? - According to you, are there foods that you should eat to promote muscle growth and strength when doing weight training?  1. When you are exercising regularly, to what extent do additional foods (such as between meal snacks, protein shakes and energy bars) play a role in your health or your development of fitness or strength?   Potential prompt if protein supplements are mentioned by the participants:   - To what extent do high-protein foods and protein supplements provide benefits to your strength gains during training? Why/why not?   Potential prompt if the low-carb, high fat diet is mentioned by participants:   - To what extent does the low-carb, high fat diet provide benefits to your fitness development? |
| **Perceived barriers and enablers** |
| 1. How does your current training program or lifestyle impact on the way you eat?   Potential prompts:   - Why? - And why not?  1. What gets in the way of you following a diet that is optimal for your health, your ability to perform at your best and your recovery during your training?   Potential prompts:   - How so?   Potential prompt if food quality or catering is mentioned by participants:   - Let’s talk about food quality/catering. Can you tell me how this impacts on your ability to follow a diet that is optimal for your health and performance?  1. Let’s talk about time during meal breaks. Please tell me whether you have enough time, too much or not enough time.   Potential prompts:   - Why? - Why not?  1. Please tell me whether you have enough, too much, or not enough time to eat snacks during your training?   Potential prompts:   - Why? - Why not?  1. What, if anything, prevents you from having an optimal diet before, during and/or after physical activity during your training program?   Potential prompts:   - How so?  1. According to you, how difficult is it to eat enough to satisfy your hunger during your training program?   Potential prompts:   - What stops you from eating more during your training? - How does the level of physical activity you do impact on your hunger levels?  1. What factors, if any, make it difficult to eat or prepare nutritious food outside of the mess – such as in the evenings and on the weekends?   Potential prompts:   - How does that impact on your ability to follow an optimal diet for your health and performance?  1. What would be the benefits of providing Infantry trainees with additional foods or foods specifically designed for their needs?   Potential prompts:   - Would there be any benefit of providing Infantry trainees with additional high-protein foods or protein supplements? - How well would they be received?  1. What challenges would you foresee if Infantry trainees were provided with supplements (for example, protein supplements)?   Potential prompts:   - How well would they be received? - To what extent would they be used? |
| **Self-efficacy** |
| 1. What do you know about carbohydrate?   Potential prompts:   - Considering the amount of physical activity you do, and your energy needs, what do you know about carbohydrate?  1. Where does your knowledge about nutrition, such as protein or carbohydrate, come from? 2. What are your sources of information about nutrition? 3. What information have you come across since being in army about nutrition? 4. Do you think army provide enough guidance on how to eat to perform at your best, or not enough? 5. What are some of the recommendations you’ve been given regarding eating and performance since being in the Army? 6. What do you know about protein, considering the amount of physical activity you do? 7. To what extent do you feel confident in your ability to follow a diet that is “balanced” or provides “optimal nutrition” during your training program?   Potential prompts:   - Why is that so? - Can you tell me more? - How confident are you that you can meet all your nutritional needs during your current training? - Does anyone feel differently about their ability to follow a balanced diet?  1. Thinking about performance, to what extent do you feel confident that you can follow a diet providing “optimal nutrition” for performing at your best during physical activity?   Potential prompts:   - Does anyone feel differently? - What are others’ thoughts? - To what extent do you feel confident in your ability to choose foods that provide enough energy (or ‘carbohydrate’) for the amount of exercise you do? - To what extent do you feel confident that you eat enough carbohydrate to perform at your best during exercise/physical activity? - To what extent do you feel confident that you can follow a diet providing “optimal nutrition” for your health during your training?  1. Thinking about recovery, to what extent do you feel confident in your ability to follow an “optimal diet” for recovery from physical activity?   Potential prompts:   - Does anyone feel differently? - To what extent do you feel confident that you eat sufficient carbohydrate for recovery from physical activity?   To what extent do you feel confident that you eat sufficient protein for recovery and muscle growth after physical activity? |
| **Cues to action** |
| 1. What makes you want to eat in a better way?   Potential prompts:   - What has made you change the way you eat in the past? - What is a recommendation or advice given by someone to you about nutrition? Did it make you change your diet? What made it effective in changing your diet? Or if it wasn’t effective advice, what made you decide not to follow the recommendation? - What else has helped you change in the past?  1. What do you think motivates Infantry trainees or soldiers to change the way they eat or take supplements?   Potential prompts:   - What makes Infantry trainees start eating for better health or fitness? |

To ensure all participants’ views are captured on each question, on each occasion that a new topic is raised by a participant, and/or when some participants haven’t had the opportunity to express their thoughts on a topic, the moderator will seek to determine whether there is consensus or differences in opinion among the group. This will be done by asking the following questions (or similar) as appropriate:

- What are others’ thoughts on this topic?
- Does anyone else have anything to add?
- Does anyone have a different opinion?
- Who has a different understanding? What are your thoughts on this topic?
